# Dysfunctional maltreatment-related cognitions in children and adolescents

**DOI:** 10.1186/s13034-017-0168-1

**Published:** 2017-06-25

**Authors:** Anke de Haan, Helene G. Ganser, Annika Münzer, Andreas Witt, Lutz Goldbeck

**Affiliations:** 1 Department of Child and Adolescent Health Psychology, University of Zurich, Zurich, Switzerland; 2 Department of Child and Adolescent Psychiatry and Psychotherapy, University Hospital Ulm, Ulm, Germany

**Keywords:** Dysfunctional cognitions, Maltreatment, Multiple linear regression analysis, Psychopathology

## Abstract

**Background:**

Dysfunctional trauma-related cognitions correlate highly with chronic stress. Studies on maltreatment-related cognitions and their predictors in children and adolescents are rare.

**Methods:**

The study sample consisted of 231 children aged 8–17 years who had experienced maltreatment including domestic violence, emotional abuse, neglect, physical, and sexual abuse. Using multiple linear regression analysis, gender, age, index-event, multi-type maltreatment, out-of-home-care, and migration background were investigated as possible predictors of dysfunctional maltreatment-related cognitions. Additionally, the associations between dysfunctional cognitions and posttraumatic stress symptoms (PTSS) as well as further internalizing and externalizing symptoms were calculated.

**Results:**

Gender emerged as a significant predictor of dysfunctional maltreatment-related cognitions. Moreover, there was an interaction effect of gender and age, with female adolescents showing most dysfunctional cognitions. Furthermore, experiencing five different maltreatment types had an impact, leading to more dysfunctional cognitions compared to single-type maltreatment. Dysfunctional maltreatment-related cognitions correlated highly with PTSS and internalizing symptoms, and moderately with externalizing symptoms.

**Conclusions:**

Dysfunctional maltreatment-related cognitions are associated with psychological symptoms after maltreatment and, therefore, need to be addressed in assessment and treatment.

*Trial registration* DRKS00003979. Registered 03 July 2012

## Background

Child maltreatment is associated with an increased risk of long-persisting mental and physical problems [1–4] including cognitive aspects such as negative self-associations [5, 6]. Caregivers and other important persons are often involved in maltreatment which can have a dramatic impact on a child’s view of himself, his family, and the world.

Cognitive models from trauma research might be helpful in understanding the impact of cognitions on maltreatment recovery. One recognized trauma model is Ehlers and Clark’s cognitive model of posttraumatic stress disorder [7]. It suggests that appraising the traumatic event and its consequences as extremely negative leads to a feeling of current threat with external-related thoughts such as “the world is a scary place where I am highly vulnerable” and internal-related thoughts such as “I am an incompetent person, I will never be the same again”. This perception of current threat is accompanied by intrusions and symptoms of arousal, anxiety, and other emotional responses. Moreover, it also motivates behavioral and cognitive responses which are intended to reduce perceived threat and distress for a short period of time. However, they have the long-term consequence of preventing cognitive change and, therefore, of maintaining the disorder [7]. Permanent and extremely negative appraisals about oneself and the world is conceptualized in the posttraumatic stress disorder (PTSD) symptom cluster *negative alterations in cognitions and mood* within the latest edition of *the Diagnostic and Statistical Manual of Mental Disorders* (DSM-5) [8].

In line with Ehlers and Clark’s model, a lot of research has been done focusing on the extremely negative appraising of the trauma and its consequences. These trauma-related cognitions, also called dysfunctional posttraumatic cognitions, were investigated in heterogeneous international trauma studies showing significant correlations between dysfunctional posttraumatic cognitions and acute stress disorder [9, 10], PTSD [11–13], symptoms of depression and anxiety [14, 15] as well as externalizing symptoms [14]. Much of the above mentioned research was conducted in samples of children and adolescents with single or accidental traumatic experiences. Studies involving children and adolescents who have been exposed to chronic stress exposure, e.g. maltreatment are rare. Leeson and Nixon [16] had a small sample of children who had experienced maltreatment (*n* = 24) and a control group (*n* = 26). They found that children's dysfunctional cognitions about permanent change and a scary world were associated with self-reported depression, self-esteem, and posttraumatic stress symptoms (PTSS). These findings are in line with other studies which found that maltreatment-related cognitions such as threat appraisal or self-blame were associated with internalizing and externalizing problems (e.g. [17, 18]). However, further studies in children and adolescents on maltreatment-related cognitions focusing on the constructs fragile person and scary world, derived from Ehlers and Clark’s model [7], are missing.

Furthermore, the cognitions’ impact on posttraumatic psychopathology is widely acknowledged, but studies on predictors of dysfunctional cognitions are rare. Investigating possible predictors might help to identify children and adolescents more vulnerable to develop and maintain dysfunctional maltreatment-related cognitions. Ehlers and Clark’s model [7] was developed for adults, but the model is applicable for children and adolescents [19, 20]. However, developmental factors should be considered, such as the child’s developmental stage including abstract cognitive abilities, the role of the family etc. [21].

Just a few studies have investigated predictors up to now: Significant gender differences were found, with girls having significantly more dysfunctional posttraumatic cognitions; but no age effect has been detected so far [15, 22]. Additionally, the effect of the trauma type experienced were investigated. Liu and Chen’s study [14] found that children and adolescents, who had experienced a traffic accident, showed most dysfunctional posttraumatic cognitions followed by participants reporting a personal trauma, medical trauma, or natural disaster. In contrast, Meiser-Stedman et al. [15] reported that children, who had experienced an assault, had significantly more dysfunctional posttraumatic cognitions than those who had experienced an accident. Palosaari, Punamäki, Peltonen, Diab and Quota [23] found that war trauma, parental psychological maltreatment, sibling conflict, and loneliness among peers predicted dysfunctional posttraumatic cognitions in war-affected children aged 10–12 years.

Further impact factors can be traced from maltreatment research in children and adolescents: Since several studies showed that experiencing multi-type maltreatment had a significant impact on symptom severity [24, 25], experiencing multi-type maltreatment might also lead to more dysfunctional maltreatment-related cognitions. Moreover, out-of-home-care might impact the amount of dysfunctional maltreatment-related cognitions as well. Kolko et al. [26] described that the prevalence of clinically significant PTSS was higher for children who were placed in out-of-home care than those maintained at home. So, there might be similar results regarding dysfunctional cognitions. Furthermore, coming from a migration background might also have an impact. Migration itself can be a very stressful [27], moreover, Schick et al. [28] found that a migration background was a risk factor of child maltreatment. Additionally, the prevalence in mental disorders differed between migrants and non-migrant in a study by Gaber et al. [29]. It might be possible that there is also a migration-specific effect on dysfunctional maltreatment-related cognitions. Additionally, variables such as socio economic status, perpetrator, and age at onset might impact developing and maintaining dysfunctional maltreatment-related cognitions. However, due to a third of children in out-home-care and mainly multi-type maltreatment in our study sample we were not able to investigate these variables.

In the current paper, we included variables which have been investigated within dysfunctional posttraumatic cognitions studies such as age, gender, and index-event. Since research on depression regarding cognitive vulnerability showed significantly different cognitive style trajectories in males and females aged between 11 and 15 leading to significantly greater cognitive vulnerability in female adolescents [30], we also investigated the interaction effect of age and gender on maltreatment-related cognitions. Unfortunately, children with a maltreatment background often experience more than one event and/or more than one type of maltreatment, e.g. physical and sexual abuse [25]. Therefore, irrespectively of multi-type maltreatment we asked the children to subjectively rate their most stressful event. Additionally, we chose variables from maltreatment research such as multi-type maltreatment, out-of-home-care, and migration background.

Furthermore, we were interested in the association between cognitions and psychopathology. As mentioned above, a lot of studies in traumatized samples showed significant associations between dysfunctional cognitions and psychopathology. However, dysfunctional cognitions correlated strongly with internalizing symptoms but only to a limited degree with externalizing symptoms suggesting that they are both of interest but should be investigated separately.

Summing up, in this study we sought to fill the current gaps in the literature on maltreatment-related cognitions in investigating the following two research questions:

First, we wanted to explore possible predictors for dysfunctional maltreatment-related cognitions. We considered gender (female > male), age at assessment (adolescents > children), interaction of gender and age, out-of-home-care (yes > no), migration background (yes > no), and multi-type maltreatment (multi-type > single-type maltreatment).

Secondly, we investigated associations of dysfunctional maltreatment-related cognitions with a range of self-reported internalizing and externalizing symptoms, and especially with self-reported PTSS. We hypothesized strong positive correlations between cognitions, PTSS, and further internalizing symptoms, as well as moderately correlations between cognitions and externalizing symptoms.

## Methods

### Procedure

We included children and adolescents with a known history of exposure to maltreatment reported by the responsible child welfare agency. All participated voluntarily in the German multi-site study *CANMANAGE,* which is a research collaborative addressing the implementation of managed mental healthcare for children and adolescents who have experienced abuse or neglect (DRKS00003979). The study was approved by the Institutional Review Boards at the different recruiting study sites. Four clinics for child and adolescent psychiatry/psychotherapy in the German federal states of Baden-Wurttemberg, North Rhine-Westphalia, and Lower Saxony served as recruiting study sites in close collaboration with child welfare institutions that referred eligible children and adolescents to the centers. Inclusion criteria were age between 4 and 17, caregivers’ willingness to participate, experience of child abuse and/or neglect as well as informed consent of all legal guardians. Taken all study sites together, 478 children and adolescents were invited for study participation, 65 were not interested in participating in a study in general, 38 declined to cooperate after they had been informed specifically about the CANMANAGE study. In total, 375 participants aged from 4 to 17 who had experienced maltreatment including domestic violence, emotional abuse, neglect, physical, and/or sexual abuse participated in the CANMANAGE project. For the current paper, 107 participants who were younger than 8 years old were not included. Due to the study design, they had not completed the self-report measures we used in our analyses. Out of these 268 eligible participants, 37 participants were excluded because of missing data in relevant variables such as dysfunctional maltreatment-related cognitions, PTSS etc. (> 25% missing data per questionnaire). This led to a sample size of 231 participants, from which 157 had been referred by child welfare institutions and 74 were recruited from clinical settings or came on their own initiative.

### Measures

#### Maltreatment

Maltreatment experiences were assessed using the German version of the structured interview *Juvenile Victimization Questionnaire* (JVQ) [31] showing good psychometric properties (Cronbach’s α = .80; κ = .59 [32]). Each child was accompanied either by his parents (*n* = 148, 64.1%) or in one-third by foster care workers or sometimes by other relatives such as grandparents (*n* = 83, 35.9%). The participating caregivers were non-offending or no longer offending. Due to the research collaborative study design, child and attendant were interviewed together. It was beneficial to have caregiver and child do the interview together, because both reports could be easily combined. However, it is possible that children might have been inhibited by the presence of parents in the interview situation. Nevertheless, in one-third of the cases the children were accompanied by foster care workers or other relatives. Furthermore, most children had been referred by child welfare institutions, therefore, their maltreatment history had been known beforehand. Additionally, separate interviews were possible if either the child or caregiver showed discomfort with the situation. If more than one episode within the JVQ was affirmed, study participants identified the “worst” or most upsetting event. We assumed that the most upsetting event might be the most impacting and relevant event at the moment. Therefore, this event was called “index-event” and referred to when assessing PTSS and dysfunctional maltreatment-related cognitions. Standardized clinical evaluation was performed by trained assessors supervised by study coordinators and the principal investigator.

#### Maltreatment-related cognitions

The German version of the *Child Posttraumatic Cognitions Inventory* (CPTCI) [15] is a self-report measure for children and adolescents assessing dysfunctional trauma-related cognitions, derived from Ehlers and Clark’s model [7]. The two subscales consist of 13 items for the subscale *permanent and disturbing change* (CPTCI-PC) and 12 items for the subscale *fragile person in a scary world* (CPTCI-SW), which are rated on a 4-point scale with 1 (*don’t agree at all*), 2 (*don’t agree a bit*), 3 (*agree a bit*), and 4 (*agree a lot*). The scores range from 25 to 100 for the total scale, from 13 to 52 for subscale CPTCI-PC, and from 12 to 48 for subscale CPTCI-SW. Examples for items are “My reactions since the frightening event mean I have changed for the worse” (CPTCI-PC item) or “I can’t stop bad things from happening to me” (CPTC-SW item). The German version showed good psychometric properties in both total scale (*Cronbach*’s α = .94) and subscales (*Cronbach*’s α = .91 and .86) [12]. Since the subscales were highly correlated with the total score (Spearman’s correlations = .94 and .93, *p* < .001) as well as highly intercorrelated (Spearman’s correlations = .76, *p* < .001) in our current sample, only the total score was used (*Cronbach*’s α = .92).

#### Posttraumatic stress symptoms

The German version of the *University of California at Los Angeles Post*-*Traumatic Stress Disorder Reaction Index* (UCLA PTSD-RI) [33] is a self-report measure of PTSS according to DSM-IV for school-age children and adolescents with good psychometric properties (e.g. *Cronbach*’s α = .88–.91 [34]; current study *Cronbach*’s α = .83). For the total score 17 items were included, rated on a 5-point scale from 0 (*none of the time*) to 4 (*most of the time*).

#### Internalizing and externalizing symptoms

Internalizing and externalizing symptoms were assessed using the self-report of the German version of the *Strengths and Difficulties Questionnaire* (SDQ) [35]. This 25-item questionnaire rated on a 3-point scale with 0 (*not true*), 1 (*somewhat true*), and 2 (*certainly true*) showed adequate psychometric properties (*Cronbach*’s α mean = .73) [36]. Although the self-report version was developed for children aged 11–17 years old, we used it for our whole sample including children aged 8–10 years. Mellor [37] showed that the self-report is applicable for this younger age group. The measure has five subscales consisting of five items each: prosocial behavior, conduct problems, peer relationship problems, emotional problems, and hyperactivity/inattention. We did not include the prosocial subscale, but used the other four subscales (= 20 items) to create a total difficulties score (*Cronbach*’s α = .77). On a sub-score level [38], the subscales peer relationship problems and emotional problems were subsumed to the sub-score internalizing problems (*Cronbach*’s α = .72); the subscales conduct problems and hyperactivity/inattention to the sub-score externalizing problems (*Cronbach*’s α = .67).

### Data analyses

Statistical analyses were performed using the *Statistical Package for Social Sciences* (SPSS, version 21.0). Statistical significance was established at an alpha level of .05.

First, descriptive analysis regarding sample description and dysfunctional maltreatment-related cognitions were calculated. To get a first impression how relevant the items were, we checked how many participants rated each item with 3 (*agree a bit*) and 4 (*agree a lot*). Secondly, a multiple linear regression analysis was calculated for investigating gender, age, interaction of gender and age, index-event, multi-type maltreatment, out-of-home-care, and migration background as possible predictors. All predictors were categorical variables; therefore, effect coding was applied to them. Effect coding compares how the effect differs from the grand mean [39]. Dummy coding was applied to the variable multi-type maltreatment with subgroup single-type maltreatment used as the reference variable to compare with the other options of multi-type maltreatment ranging from two to five maltreatment types. Experiencing five types meant, for example, that these participants had experienced domestic violence, emotional abuse, neglect, physical abuse, and sexual abuse. When calculating the multiple linear regression analysis, we included all variables in one step simultaneously. Finally, Spearman’s correlations were conducted to investigate the association between cognitions, PTSS, further internalizing, and externalizing symptoms. Because of these multiple tests the *p* values were *Sidak*-adjusted in order to prevent misleading results due to alpha error inflation.

## Results

### Descriptive analyses

First of all, the description of our sample is given in Table 1.

**Table 1:** Sociodemographic data and maltreatment-related information

Variable	Subgroup	Total sample *N* = 231	
		*n*	%
Gender	Male	133	57.6
	Female	98	42.4
Age	Children (8–12)	149	64.5
(*M* = 12.0, *SD* = 2.5)	Adolescents (13–17)	82	35.5
School	Elementary school	63	27.3
	Middle and high school	106	45.9
	School for children with learning difficulties	46	19.9
	Not determined^a^	16	6.9
Household incomes per month	Under 500 €	7	3.0
	500 €–under 1000 €	26	11.3
	1000 €–under 2000 €	65	28.1
	2000 €–under 3000 €	40	17.3
	3000 €–under 4000 €	25	10.8
	4000 €–under 5000 €	20	8.7
	5000 € and more	11	4.8
	Not determined^a^	37	16.0
Occupation mother	Not employed (e.g. pensioner, student etc.)	65	28.1
	Unemployed, seeking work	35	15.2
	Temporary leave of absence e.g. parental leave	8	3.5
	Part-time job or employed on hourly basis	68	29.4
	Full-time job	34	14.7
	Apprentice	1	.4
	Not determined^a^	20	8.7
Occupation father	Not employed (e.g. pensioner, student etc.)	29	12.6
	Unemployed, seeking work	22	9.5
	Temporary leave of absence e.g. parental leave	0	.0
	Part-time job or employed on hourly basis	16	6.9
	Full-time job	112	48.5
	Apprentice	3	1.3
	Not determined^a^	49	21.2
Index-event	Domestic violence	57	24.7
	Emotional abuse	23	10.0
	Neglect	30	13.0
	Physical abuse	65	28.1
	Sexual abuse	56	24.2
Maltreatment type (*Note.* Take multi-type maltreatment in account)	Domestic violence	158	68.4
	Emotional abuse	124	53.7
	Neglect	131	56.7
	Physical abuse	175	75.8
	Sexual abuse	89	38.5
Co-occurrence of maltreatment types	Single-type maltreatment	32	13.9
	Two types	58	25.1
	Three types	59	25.5
	Four types	58	25.1
	Five types	24	10.4
Out-of-home-care	Yes	78	33.8
	No	153	66.2
Migration background^b^	Yes	75	32.5
	No	128	55.4
	Not determined^a^	28	12.1

^a^“Not determined” means that these participants could not be reliably classified in any category due to insufficient information

^b^Migration background was defined as non-German nationality or non-German place of birth of the child or at least of one parent

Table 2 shows the five dysfunctional maltreatment-related cognitions the participants agreed the most with. It included thoughts such as “I can’t stop bad things from happening to me”, “Anyone could hurt me”, or “I’m scared that I’ll get so angry that I’ll break something or hurt someone”.

**Table 2:** Top 5 dysfunctional maltreatment-related cognitions

Item	Agree a bit/agree a lot (%)	Subscale
I can’t stop bad things from happening to me	50.2	CPTCI-SW
Anyone could hurt me	41.1	CPTCI-SW
I’m scared that I’ll get so angry that I’ll break something or hurt someone	36.8	CPTCI-PC
I can’t cope when things get tough	35.9	CPTCI-SW
I have to watch out for danger all the time	32.0	CPTCI-SW

*N* = 231

*CPTCI-PC* subscale child post-traumatic cognitions inventory permanent and disturbing change, *CPTCI-SW* subscale child post-traumatic cognitions inventory fragile person in a scary world

### Possible predictors

Table 3 shows means, standard deviations, minima and maxima of dysfunctional maltreatment-related cognitions.

**Table 3:** Means, standard deviations, and ranges of dysfunctional maltreatment-related cognitions

Variable	*n*		CPTCI total score range (25–100)			
			*M*	*SD*	Min	Max
Gender	133	Male	42.84	13.12	25	86
	98	Female	48.32	15.40	25	95
Age (at assessment)	149	Children (8–12)	44.05	13.00	25	86
	82	Adolescents (13–17)	47.20	16.44	25	95
Male	91	Children (8–12)	44.09	13.47	25	86
	42	Adolescents (13–17)	40.14	12.04	25	75
Female	58	Children (8–12)	43.98	13.33	25	76
	40	Adolescents (13–17)	54.60	17.30	25	95
Index-event	57	Domestic violence	43.51	12.98	25	78
	23	Emotional abuse	43.96	16.15	25	84
	30	Neglect	45.20	14.07	26	86
	65	Physical abuse	43.68	12.64	25	86
	56	Sexual abuse	49.05	16.56	25	95
Co-occurrence of maltreatment types	32	Single-type maltreatment	43.25	16.41	25	90
	58	Two types	44.16	13.14	25	84
	59	Three types	42.95	13.19	25	86
	58	Four types	45.72	12.28	25	76
	24	Five types	54.25	18.74	28	95
Out-of-home-care	78	Yes	43.78	12.19	25	86
	153	No	45.87	15.33	25	95
Migration background^a^	75	Yes	44.23	13.21	25	90
*N* = 203	128	No	44.63	14.79	25	95

Sample size *N* = 231, except migration background

*CPTCI* child post-traumatic cognitions inventory

^a^Migration background was defined as non-German nationality or non-German place of birth of the child or at least of one parent

Gender, the interaction of gender and age as well as experiencing all kind of maltreatment types (co-occurrence of all five maltreatment types) had a significant impact on dysfunctional maltreatment-related cognitions (see Table 4). The overall model explained 20% of the variance in dysfunctional maltreatment-related cognitions (*F*(13) = 3.63, *p* < .001).

**Table 4:** Predictors of dysfunctional maltreatment-related cognitions

	Unstandardized coefficients		Standardized coefficients		
	*Β*	*SE B*	β	*t*	*p*
Constant	40.80	2.76		14.81	.000
Gender	3.48	1.02	.24	3.40	.001
Age (at assessment)	1.62	1.02	.11	1.58	.115
Gender × age	4.46	1.01	.31	4.40	.000
Index-event					
Domestic violence	.71	1.80	.03	.39	.695
Neglect	.63	2.29	.02	.28	.782
Physical abuse	−.78	1.72	-.03	−.45	.652
Sexual abuse	1.58	1.91	.06	.83	.410
Co-occurence^a^					
Two types	3.16	3.26	.10	.97	.334
Three types	1.44	3.29	.04	.44	.662
Four types	2.53	3.21	.08	.79	.433
Five types	11.19	3.95	.24	2.84	.005
Out-of-home-care	−1.68	1.06	−.11	−1.58	.116
Migration background^b^	−.35	.99	−.02	−.35	.725
Model summary	*F*(13) = 3.63, *p* < .001, *R* = .447, *R* ^*2*^ = .200, *R* ^*2*^ adj. = .145				

Sample size *N* = 203

*SE* standard error

^a^Subgroup single-type maltreatment was used as the reference variable for testing the impact of co-occurrence of maltreatment types

^b^Migration background was defined as non-German nationality or non-German place of birth of the child or at least of one parent

### Association with psychological symptoms

Dysfunctional maltreatment-related cognitions correlated strongly (*r* > .50) with PTSS, further internalizing symptoms as well as the total difficulties score. They were moderately associated (*r* > .30) with externalizing symptoms (see Table 5). The correlations between cognitions and PTSS were significantly stronger than the correlation between cognitions and externalizing symptoms (*r* = .72 vs. *r* = .43, *Z* = 4.78, *p* < .001). Furthermore, the association between cognitions and internalizing symptoms were also significantly stronger than cognitions and externalizing symptoms (*r* = .65 vs. *r* = .43, *Z* = 3.37, *p* < .001).

**Table 5:** Spearman’s correlations between dysfunctional maltreatment-related cognitions, posttraumatic stress symptoms, further internalizing, and externalizing symptoms

	UCLA PTSD-RI	SDQ internalizing problems	SDQ externalizing problems	SDQ total difficulties score
CPTCI total score	.72	.65	.43	.64

Sample size *N* = 231

*CPTCI* child post-traumatic cognitions inventory, *UCLA PTSD-RI* University of California at Los Angeles Post-Traumatic Stress Disorder Reaction Index, *SDQ* Strength and Difficulty Questionnaire

*p* values were Sidak-adjusted. They were all significant at a *p* < .001 level

## Discussion

The aim of our study was to gain better understanding of dysfunctional maltreatment-related cognitions in children and adolescents by investigating possible predictors of dysfunctional cognitions as well as their correlations with internalizing and externalizing symptoms. First of all, we found that dysfunctional cognitions regarding permanent and disturbing change and fragile person in a scary world derived from Ehlers and Clark’s model [7] were relevant in children and adolescents with a chronic maltreatment background: For example, 50% of our sample agreed with the thought “I can’t stop bad things from happening to me” portraying a feeling of a fragile person in a scary world. Furthermore, on total scale level, we found descriptively that the means of dysfunctional maltreatment-related cognitions in the subgroups females, adolescents, female adolescents, index-event sexual abuse, and experiencing all five maltreatment types were within and above the clinically significant CPTCI cutoff range of 46 to 48. This cutoff range was found to be the best indicator of clinically significant appraisals determined by the presence of PTSD in a hospital-recruited sample of 535 participants aged 7–17 years [40]. Although the differences in the sample background needs to be taken into account, it shows that we had dysfunctional maltreatment-related cognitions within a clinical relevant range in our sample.

Consistent with this descriptive observation mentioned above, gender as well as the interaction of gender and age were significant predictors for dysfunctional maltreatment-related cognitions. In line with previous studies [15, 22], girls had significantly more dysfunctional maltreatment-related cognitions than boys; age did not have a significant effect. Building on these findings of previous studies, a significant interaction effect of gender and age was detected, with female adolescents showing most dysfunctional cognitions. This is in line with Mezulis et al. [30] who described that significantly different cognitive style trajectories in males and females aged between 11 and 15 led to significantly greater cognitive vulnerability in female adolescents. Two depression research models might help to understand these differences in adolescents better. Research findings who support the *exposure model* reported that a higher prevalence of depression in female adolescents [41] can be explained by a higher cognitive vulnerability in females [42, 43]. However, a second model, the *cognitive scar model* [44], suggests that preceding higher depression scores in girls predict higher dysfunctional cognitions in female adolescents [30]. Adapting these two models to dysfunctional maltreatment-related (or posttraumatic) cognitions and PTSD might help us to understand the association between age, gender, cognitions, PTSS, and depression better. Different ways are possible: (a) The reason for a higher female PTSD prevalence after traumatic events [45] might emerge due to higher levels of dysfunctional cognitions in females; (b) Preceding higher PTSD scores in girls might predict higher dysfunctional cognitions in female adolescents; (c) Preceding higher depression scores in girls might predict higher dysfunctional cognitions which eventually lead to higher PTSD scores in female adolescents. Longitudinal research on the transition from childhood to adolescence is needed. However, when investigating age and gender it might be important to take the trauma experience in account. Female participants often report more sexual abuse than male participants [46]. In our sample, female adolescents reported more sexual abuse (57.5%) than female children (46.6%), male adolescents (35.7%), or male children (26.4%). Regarding sexual abuse as index-event we found the following distributions: female adolescents 40%, female children 27.6%, male adolescents 26.2%, and male children 14.3%. However, we did not find that sexual abuse (as index-event) or any other maltreatment type was significantly associated with more dysfunctional cognitions compared to the other maltreatment types. This might be explained by the fact that even if their subjectively most stressful event differed, most of them had a similar history of multiple forms of maltreatment. Regarding multi-type maltreatment, we only found a significant impact of experiencing all five kind of maltreatment types (domestic violence, emotional abuse, neglect, physical abuse, and sexual abuse) on cognitions compared to single-type maltreatment. Experiencing two to four maltreatment types compared to single-type maltreatment did not predict significantly more dysfunctional cognitions. This is contrary to several studies showing that experiencing multi-type maltreatment had a significant impact on symptom severity [24, 25]. Dysfunctional cognitions and symptoms severity might, therefore, be seen independently in the context of multi-type maltreatment, even though they are generally highly correlated. Further studies are needed to understand the association between multi-type maltreatment, dysfunctional cognitions, and symptom severity. Furthermore, neither out-of-home-care nor migration background emerged as significant predictors for dysfunctional maltreatment-related cognitions. The latter is in line with studies about other variables such as health-related quality of life [47] and internalizing disorders [48] that did not find significant differences between migrants and non-migrants either. All in all, our regression model only accounted for 20% variance and when adjusted to the amount of variables we were using only 14.5%. Focusing solely on child-related cognitions seems not enough.

Regarding our second hypothesis the association between dysfunctional maltreatment-related cognitions and psychopathology our findings of strong correlations between dysfunctional maltreatment-related cognitions and self-reported PTSS as well as further internalizing symptoms are consistent with previous studies [12, 15, 16]. In contrast, studies comparing dysfunctional cognitions with parent-reported internalizing problems found smaller correlations [14, 16]. Discrepancies between self-reports and proxy-reports are in line with the literature [49]. The assessment of internalizing problems preferably includes multiple informants [50–54], because parents might underestimate symptoms [55] or their reporting might be influenced by their own symptoms [56]. Despite the differences between self-reports and proxy-reports, the strong correlations between dysfunctional maltreatment-related cognitions and self-reported internalizing symptoms support Leeson and Nixon’s statement [16] that dysfunctional cognitions play a particularly important role in the development of internalizing symptoms in children who have experienced maltreatment, similar to adult studies [6, 57, 58]. However, the relationship between dysfunctional maltreatment-related cognitions, PTSS, and internalizing symptoms could also be comparable to the results about cognitive vulnerability in depression which reported bidirectional changes over time among cognitions and internalizing symptoms [59]. Due to our cross-sectional design we were not able to investigate causal relationships between cognitions and psychopathology.

The moderate correlations between dysfunctional cognitions and externalizing symptoms in our study were slightly stronger than in studies which had assessed proxy-reported externalizing symptoms [14, 16]. Again, discrepancies between self-reports and proxy-reports might be one reason for the slightly different results. However, other studies showed that child maltreatment had the long-term effect of externalizing and antisocial behavior impacted by objective variables such as chronicity [60] as well as more subjectively biased variables such as alienation from the primary caregiver [61]. Dysfunctional maltreatment-related cognitions might therefore be a basis for later externalizing behaviour problems. One-third in our sample agreed with the thought “I’m scared that I’ll get so angry that I’ll break something or hurt someone” which might give an idea about the relevance of externalizing problems. Further studies on trauma, maltreatment, dysfunctional cognitions, and externalizing symptoms are needed to gain a better understanding of their relationships. Additionally, gender-specific pattern should be taken into account: Cullerton-Sen et al. [62], for example, reported that maltreatment was associated with physical aggression for male adolescents and relational aggression for female adolescents.

### Limitations

Several limitations of this study need to be mentioned. Because of the cross-sectional design, we could not prove any causal relationship between variables. Nevertheless, we were able to add important information regarding dysfunctional cognitions and psychological symptoms from a cross-sectional angle. Moreover, we only used child self-reported data regarding dysfunctional cognitions and psychopathology which might have led to a biased information content. Further limitations emerge from our study sample, because most participants reported multi-type maltreatment. Multi-type maltreatment is very common in children and adolescents with a maltreatment background. However, it made it difficult for us to assess the impact of distinct maltreatment-types or timing of abuse. Additionally, we were not able to control for variables such as socio economic status, perpetrator, and age at onset which might have had an effect as well. They might be the reason for non-significant findings regarding maltreatment type or frequency. Maybe perpetrator and age at onset play a more important role than the event itself or they interact with the event and frequency. Furthermore, our study focused solely on demographic variables, however, variables such as temperament or coping styles might also play an important role.

### Implications

The strong associations between dysfunctional maltreatment-related cognitions and psychological symptoms underline the clinical relevance of dysfunctional appraisals which should be included routinely in clinical assessment. With the CPTCI original and short form [40], two screening instruments are available for children and adolescents to assess and monitor dysfunctional cognitions systematically. Female adolescents, in particular, tend to develop dysfunctional maltreatment-related cognitions, and this need to be taken into account when planning interventions. Cognitive restructuring in the case of distorted maltreatment-related cognitions might, therefore, be a promising strategy to prevent chronic psychological problems following victimization, something that has already been demonstrated for cognitive treatments of maltreatment-related posttraumatic stress disorder [63].

Furthermore, dysfunctional maltreatment-related cognitions are associated not only with internalizing, but also externalizing symptoms. The relationship between dysfunctional cognitions and externalizing symptoms merits more attention in research and clinical practice. Otherwise, maltreated children with externalizing symptomatic might be treated only on the behavioral level and the underlying cognitive component might be missed.

Further research is needed on different topics. On one hand, more research is needed to understand gender differences for developing dysfunctional cognitions in the aftermath of maltreatment and/or trauma. Additionally, other possible predictors of dysfunctional posttraumatic or maltreatment-related cognitions should be investigated, e.g. parental-related variables or further child-related variables such as temperament, coping styles, and cognitive ability. Structural equation modeling combining predictors, dysfunctional cognitions, and child symptoms in a longitudinal design would be a further step. Furthermore, more longitudinal studies are necessary to investigate the pathways between cognitions and psychopathology. Especially research about dysfunctional cognitions and externalizing symptoms are of interest.

## Conclusions

Dysfunctional maltreatment-related cognitions have been rarely investigated so far, therefore, we are able to add important knowledge to this topic with the findings from our large study sample. Both the descriptive analysis of the dysfunctional maltreatment-related cognitions and their strong associations with psychological symptoms underline that dysfunctional cognitions regarding permanent and disturbing change and fragile person in a scary world, derived from Ehlers and Clark’s recognized cognitive model [7], seems to be relevant in children and adolescents with a chronic maltreatment background. Due to the cognitions' association with psychopathology, dysfunctional maltreatment-related cognitions need to be addressed in assessment and treatment. Especially female adolescents tend to develop dysfunctional maltreatment-related cognitions, and this is important to keep in mind when supporting them to cope with their maltreatment experiences.

## Abbreviations

CPTCI: Child Posttraumatic Cognitions Inventory
CPTCI-PC: subscale CPTCI permanent and disturbing change
CPTCI-SW: subscale CPTCI fragile person in a scary world
DSM: Diagnostic and Statistical Manual of Mental Disorders
JVQ: Juvenile Victimization Questionnaire
PTSD: posttraumatic stress disorder
PTSS: posttraumatic stress symptoms
SDQ: Strengths and Difficulties Questionnaire
SPSS: Statistical Package for Social Sciences
UCLA PTSD-RI: University of California at Los Angeles Post-Traumatic Stress Disorder Reaction Index

## References

[1] Buckingham ET, Daniolos P (2013). Longitudinal outcomes for victims of child abuse. Curr Psychiatry Rep.

[2] Fergusson DM, Boden JM, Horwood LJ (2008). Exposure to childhood sexual and physical abuse and adjustment in early adulthood. Child Abuse Negl.

[3] Sachs-Ericsson N, Kendall-Tackett K, Hernandez A (2007). Childhood abuse, chronic pain, and depression in the National Comorbidity Survey. Child Abuse Negl.

[4] Springer KW, Sheridan J, Kuo D, Carnes M (2007). Long-term physical and mental health consequences of childhood physical abuse: results from a large population-based sample of men and women. Child Abuse Negl.

[5] Alloy LB, Abramson LY, Smith JM, Gibb BE, Neeren AM (2006). Role of parenting and maltreatment histories in unipolar and bipolar mood disorders: mediation by cognitive vulnerability to depression. Clin Child Fam Psych.

[6] van Harmelen A-L, de Jong PJ, Glashouwer KA, Spinhoven P, Penninx BWJH, Elzinga BM (2010). Child abuse and negative explicit and automatic self-associations: the cognitive scars of emotional maltreatment. Behav Res Ther.

[7] Ehlers A, Clark D (2000). A cognitive model of posttraumatic stress disorder. Behav Res Ther.

[8] American Psychological Association (2013). Diagnostic and statistical manual of mental disorders: DSM-5.

[9] Salmon K, Sinclair E, Bryant RA (2007). The role of maladaptive appraisals in child acute stress reactions. Br J Clin Psychol.

[10] Salmond CH, Meiser-Stedman R, Glucksman E, Thompson P, Dalgleish T, Smith P (2011). The nature of trauma memories in acute stress disorder in children and adolescents. J Child Psychol Psychiatry.

[11] Bryant RA, Salmon K, Sinclair E, Davidson P (2007). A prospective study of appraisals in childhood posttraumatic stress disorder. Behav Res Ther.

[12] de Haan A, Petermann F, Meiser-Stedman R, Goldbeck L (2016). Psychometric properties of the German version of the Child Post-Traumatic Cognitions Inventory (CPTCI-GER). Child Psychiat Hum Dev.

[13] Meiser-Stedman R, Dalgleish T, Glucksman E, Yule W, Smith P (2009). Maladaptive cognitive appraisals mediate the evolution of posttraumatic stress reactions: a 6-month follow-up of child and adolescent assault and motor vehicle accident survivors. J Abnorm Psychol.

[14] Liu S-T, Chen S-H (2015). A community study on the relationship of posttraumatic cognitions to internalizing and externalizing psychopathology in Taiwanese children and adolescents. J Abnorm Child Psych.

[15] Meiser-Stedman R, Smith P, Bryant R, Salmon K, Yule W, Dalgleish T, Nixon RDV (2009). Development and validation of the Child Post-Traumatic Cognitions Inventory (CPTCI). J Child Psychol Psychiatry.

[16] Leeson FJ, Nixon RDV (2011). The role of children’s appraisals on adjustment following psychological maltreatment: a pilot study. J Abnorm Child Psychol.

[17] Fosco GM, Grych JH (2008). Emotional, cognitive, and family systems mediators of children’s adjustment to interparental conflict. J Fam Psychol.

[18] Jouriles EN, Spiller CL, Stephens N, McDonald R, Swank P (2000). Variability in adjustment of children of battered women: the role of child appraisals of interparent conflict. Cognit Ther Res.

[19] Ehlers A, Mayou RA, Bryant B (2003). Cognitive predictors of posttraumatic stress disorder in children: results of a prospective longitudinal study. Behav Res Ther.

[20] Stallard P (2003). A retrospective analysis to explore the applicability of the Ehlers and Clark (2000) Cognitive Model to explain PTSD in children. Behav Cogn Psychoth.

[21] Meiser-Stedman R (2002). Towards a cognitive–behavioral model of PTSD in children and adolescents. Clin Child Fam Psych.

[22] Diehle J, de Roos C, Meiser-Stedman R, Boer F, Lindauer RJL (2015). The Dutch version of the Child Posttraumatic Cognitions Inventory: validation in a clinical sample and a school sample. Eur J Psychotraumatol.

[23] Palosaari E, Punamäki R-L, Peltonen K, Diab M, Quota S (2016). Negative social relationships predict posttraumatic stress symptoms among war-affected children via posttraumatic cognitions. J Abnorm Child Psychol.

[24] Arata CM, Langhinrichsen-Rohling J, Bowers D, O’Farrill-Swails L (2005). Single versus multi-type maltreatment. J Aggress Maltreat Trauma.

[25] Salazar AM, Keller TE, Courtney ME (2011). Understanding social support’s role in the relationship between maltreatment and depression in youth with foster care experience. Child Maltreatment.

[26] Kolko DJ, Hurlburt MS, Zhang J, Barth RP, Leslie LK, Burns BJ (2010). Posttraumatic stress symptoms in children and adolescents referred for child welfare investigation: a national sample of in-home and out-of-home care. Child Maltreatment.

[27] Bhugra D (2004). Migration and mental health. Acta Psychiat Scand.

[28] Schick M, Schönbucher V, Landolt MA, Schnyder U, Xu W, Maier T, Mohler-Kuo M (2016). Child maltreatment and migration: a population-based study among immigrant and native adolescents in Switzerland. Child Maltreatment.

[29] Gaber TJ, Bouyrakhen S, Herpertz-Dahlmann B, Hagenah U, Holtmann M, Freitag CM, Wöckel L, Poustka F, Zepf FD (2013). Migration background and juvenile mental health: a descriptive retrospective analysis of diagnostic rates of psychiatric disorders in young people. Glob Health Action.

[30] Mezulis AH, Funasaki KS, Charbonneau AM, Hyde JS (2010). Gender differences in the cognitive vulnerability-stress model of depression in the transition to adolescence. Cogn Ther Res.

[31] Hamby SL, Finkelhor D, Ormrod RK, Turner HA (2004). The Juvenile Victimization Questionnaire (JVQ): administration and scoring manual.

[32] Finkelhor D, Hamby SL, Ormrod R, Turner H (2005). The Juvenile Victimization Questionnaire: reliability, validity, and national norms. Child Abuse Negl.

[33] Steinberg AM, Brymer MJ, Decker KB, Pynoos RS (2004). The University of California at Los Angeles Post-Traumatic Stress Disorder Reaction Index. Curr Psychiatry Rep.

[34] Steinberg AM, Brymer MJ, Kim S, Briggs EC, Ippen CG, Ostrowski SA, Gully KJ, Pynoos RS (2013). Psychometric properties of the UCLA PTSD reaction index: part I. J Trauma Stress.

[35] Goodman R (1997). The Strengths and Difficulties Questionnaire: a research note. J Child Psychol Psychiatry.

[36] Goodman R (2001). Psychometric properties of the Strengths and Difficulties Questionnaire. J Am Acad Child Adolesc Psychiatry.

[37] Mellor D (2004). Furthering the use of the Strengths and Difficulties Questionnaire: reliability with younger child respondents. Psychol Assess.

[38] Goodman A, Lamping DJ, Ploubidis GB (2010). When to use broader internalising and externalising subscales instead of the hypothesised five subscales on the Strengths and Difficulties Questionnaire (SDQ): data from British parents, teachers and children. J Abnorm Child Psychol.

[39] Eid M, Gollwitzer M, Schmitt M (2011). Statistik und Forschungsmethoden.

[40] McKinnon A, Smith P, Bryant R, Salmon K, Yule W, Dalgleish T, Dixon C, Nixon RD, Meiser-Stedman R (2016). An update on the clinical utility of the Children’s Post-Traumatic Cognitions Inventory. J Trauma Stress.

[41] Hoffmann F, Petermann F, Glaeske G, Bachmann CJ (2012). Prevalence and comorbidities of adolescent depression in Germany. Z Kinder Jug-Psychol.

[42] Calvete E, Cardeñoso O (2005). Gender differences in cognitive vulnerability to depression and behavior problems in adolescents. J Abnorm Child Psychol.

[43] Hankin BL, Abramson LY (2002). Measuring cognitive vulnerability to depression in adolescence: reliability, validity, and gender differences. J Clin Child Adolesc Psychol.

[44] Nolen-Hoeksema S, Girgus JS, Seligman ME (1992). Predictors and consequences of childhood depressive symptoms: a 5-year longitudinal study. J Abnorm Psychol.

[45] Alisic E, Zalta AK, van Wesel F, Larsen SE, Hafstad GS, Hassanpour K, Smid GE (2014). Rates of post-traumatic stress disorder in trauma-exposed children and adolescents: meta-analysis. Br J Psychiatry.

[46] Tolin DF, Foa EB, Kimerling R, Ouimette P, Wolfe J (2002). Gender and PTSD: a cognitive model. Gender and PTSD.

[47] Ravens-Sieberer U, Erhart M, Wille N, Bullinger M, the BELLA study group (2008). Health-related quality of life in children and adolescents in Germany: results of the BELLA study. Eur Child Adoles Psy.

[48] Belhadj Kouider E, Lorenz AF, Dupont M, Petermann F (2015). Internalizing disorders in migrant and non-migrant children and adolescents: analyses of a German health care population. J Public Health.

[49] Achenbach TM, McConaughy SH, Howell CT (1987). Child/adolescent behavioral and emotional problems: implications of cross-informant correlations for situational specificity. Psychol Bull.

[50] Comer JS, Kendall PC (2004). A symptom-level examination of parent–child agreement in the diagnosis of anxious youths. J Am Acad Child Psychiatry.

[51] Kassam-Adams N (2006). The Acute Stress Checklist for Children (ASC-Kids): development of a child self-report measure. J Trauma Stress.

[52] Meiser-Stedman R, Smith P, Glucksman E, Yule W, Dalgleish T (2008). The posttraumatic stress disorder diagnosis in preschool- and elementary school-age children exposed to motor vehicle accidents. Am J Psychiatry.

[53] Scheeringa MS, Wright MJ, Hunt JP, Zeanah CH (2006). Factors affecting the diagnosis and prediction of PTSD symptomatology in children and adolescents. Am J Psychiatry.

[54] Silverman WK, Ollendick TH (2005). Evidence-based assessment of anxiety and its disorders in children and adolescents. J Clin Child Adolesc Psychol.

[55] Schreier H, Ladakakos C, Morabito D, Chapman L, Knudson MM (2005). Posttraumatic stress symptoms in children after mild to moderate pediatric trauma: a longitudinal examination of symptom prevalence, correlates, and parent–child symptom reporting. J Trauma.

[56] Kassam-Adams N, Garcia-Espana JF, Miller VA, Winston F (2006). Parent–child agreement regarding children’s acute stress: the role of parent acute stress reactions. J Am Acad Child Adolesc Psychiatry.

[57] Gibb BE, Benas JS, Crossett SE, Uhrlass DJ (2007). Emotional maltreatment and verbal victimization in childhood. J Emot Abuse.

[58] Valle LA, Silovsky JF (2002). Attributions and adjustment following child sexual and physical abuse. Child Maltreatment.

[59] McCarty CA, Vander Stoep A, McCauley E (2007). Cognitive features associated with depressive symptoms in adolescence: directionality and specificity. J Clin Child Adolesc Psychol.

[60] Éthier LS, Lemelin J-P, Lacharité C (2004). A longitudinal study of the effects of chronic maltreatment on children’s behavioral and emotional problems. Child Abuse Negl.

[61] Egeland B, Yates T, Appleyard K, van Dulmen M (2002). The long-term consequences of maltreatment in the early years: a developmental pathway model to antisocial behavior. Child Serv Soc Policy Res Pract.

[62] Cullerton-Sen C, Cassidy AR, Murray-Close D, Cicchetti D, Crick NR, Rogosch FA (2008). Childhood maltreatment and the development of relational and physical aggression: the importance of a gender-informed approach. Child Dev.

[63] Cohen JA, Deblinger E, Mannarino AP, Steer RA (2004). A multisite, randomized controlled trial for children with sexual abuse—related PTSD symptoms. J Am Acad Child Psychiatry.

